# Editorial

**Published:** 1897-02

**Authors:** 


### Editorial.

### A CASE OF LUST-CRUELTY.

Flannigan, who murdered two women and almost succeeded in putting an end to one man at Poplar Springs on the night of Dec. 31, under peculiarly horrifying circumstances, would appear, from the newspaper accounts of him and from his own narrative of the motive which actuated him in the commission of the deed, to be a neuropath and a sexual pervert. The special type of perverted sexual instinct to which he apparently belongs is Sadism, so named from the notorious Marquis of Sade, whose obscene novels, written about a century ago, treated of lust and cruelty, himself the subject of habitual satyriasis, with perverse sexual instinct combined with cruelty. Unnatural sexual love for a child, as was displayed in the Flannigan case, may be explained on the assumption that impotence toward adult women and fear of their contempt and derision lead him to take advantage of the child's unsophistication for the relief of his *libido sexualis.* Winning with gifts the child's confidence and affection, he was able to establish a sort of sexual unilateral mutualism with her without exciting her alarm. The discovery on the part of the little girl's parents that all was not as it should be between the man and the child, and his knowledge of their suspicions, coupled with the (to him) maddening thought of being deprived of his sole means of satisfying an imperious and dominant lust, impelled him to the commission of his atrocious act.

The wall between such perversion and insanity is thin, and the question for responsibility for his deed must be settled in court, but it is very certain that such individuals are a menace to society, and, as such, should be summarily dealt with.

### DISCONTENT.

There is an undoubted tendency at present, and probably has always been, toward unfaith in the existing order of things in medicine. Disbelief in the efficacy of drugs with some amounts almost to complete infidelity. Dr. Holmes voiced this sentiment to the fullest when he made the remark that if all physic were thrown into the sea, it would be better for men and worse for the fishes.

Fortunately such radical pessimism is not shared by all, but there is, nevertheless, with all a sense of insufficiency and incompleteness when remedy is measured against disease. This spirit, instead of being deplored, should be encouraged. Discontent is the stepping-stone to progress, and the constant stimulus toward endeavor in the attainment of perfection. It points to ampler fields beyond, to more exact results, to simpler methods,to singling out and preserving the good from the useless. Years of discontent are fat years; lean years come with complaisant satisfaction with men themselves and things. "The cankers of a calm world and a long peace."

---

### DR. GOWERS ON EMPIRICAL THERAPEUTICS.

In a recent address Dr. Gowers, in speaking of the use of drugs, claimed that the best of our still-used remedies were empirical or chance discoveries:

"We smile at the popular herbal remedies. But it is to these that we owe the majority of our most useful drugs. I can not conceive a therapeutist surveying a list of the chief drugs on which we depend in our daily work—and do not depend in vain—without a sense of wonder and perhaps of humiliation. We disinfect our rooms with burning sulphur; and so men did before the time of Homer. We purge sometimes with rhubarb, especially when some subsequent astringent influence is desirable; and so did the old Arabians for the same special reason. The value of castor oil in its chief use was familiar, probably for ages, to the natives of the East and West Indies before it was made known in Europe by a physician from Antigua one hundred an fifty years ago. Aloes was employed in the same way long before the time of Dioscorides and Pliny. The knowledge of the influence of ergot in parturition we owe to the peasants of Germany, and the use of male fern for tapeworm goes back to the old Greeks and Romans. The employment of mercury in syphilis by inunction and fumigation, which our Nineteenth century therapeutists regard with such satisfaction, seems to go back to the time of the Crusades, and it is said that its use can be traced in Malabar as far back as the Ninth century. Podophyllum as a purgative we owe to the North American Indians. If we go through the list of all the drugs on which we most rely, we find a similar story. Even in the case of those which are the latest additions to our resources, we find that, with very few exceptions, their use arose from what we must regard as pure empiricism. It was by accident that the local anesthetic influence of cocaine was discovered."—*Boston Medical and Surgical Journal.*

